# Supplementary material for: Long-read isoform sequencing reveals tissue-specific isoform expression between active and hibernating brown bears (Ursus arctos)
Source: G3 (Bethesda). 2021 Dec 21;12(3):jkab422. doi: 10.1093/g3journal/jkab422 (PMC9210309; doi:10.1093/g3journal/jkab422)
Supplement: jkab422_Supplementary_Data [file jkab422_supplementary_data.zip › Supplemental_Material.pdf]

Long-read isoform sequencing reveals tissue-specific isoform expression between active and hibernating brown bears (*Ursus arctos*)

Tables S1-S5, Figures S1-S6

Authors:
Elizabeth Tseng[1], Jason G. Underwood[1], Brandon D. Evans Hutzenbiler[2,3], Shawn Trojahn[4], Brewster Kingham[5], Olga Shevchenko[5], Erin Bernberg[5], Michelle Vierra[1], Charles T. Robbins[3,4], Heiko T. Jansen[2], Joanna L. Kelley[4,*]

Author Affiliations:
[1]Pacific Biosciences, Menlo Park, CA, USA
[2]Department of Integrative Physiology and Neuroscience, Washington State University, Pullman, WA
[3]School of the Environment, Washington State University, Pullman, WA
[4]School of Biological Sciences, Washington State University, Pullman, WA
[5]University of Delaware, Delaware Biotechnology Institute, Sequencing & Genotyping Center, Newark, DE

****Corresponding Author:***
Joanna L. Kelley, joanna.l.kelley@wsu.edu

Table S1. Number of ZMWs and FLNC per movie

See excel spreadsheet

Table S2. Stats for number of genes and transcripts for reference annotation, PacBio Iso-Seq transcriptome, and merged (PacBio and reference) transcriptome.

	**Reference**	**PacBio-only**	**Merged (PacBio+Reference)**
Number of Annotated Genes	30,263	12,018	28,124
Number of Novel Genes	N/A	907	3,705*
Total Number of Genes	30,263	12,925	31,829
Number of Transcripts	58,335	76,071	107,649
Transcript Length Range	40 – 105,000 bp	152 – 16,525 bp	40 – 105,081 bp
Mean Transcript Length	2,924 bp	3,224 bp	3,162 bp
Transcript Length 5-95% Percentile	191 – 7722 bp	1420 – 5950 bp	559 – 6871 bp

*Calculated by SQANTI3. Note that the SQANTI3 ignores reference transcripts that are less than 200 bp and as a result, the number of novel genes increased when Iso-Seq data was merged with the reference annotation.

Table S3. SQANTI3 classification of PacBio Iso-Seq and Merged transcriptome.

	**PacBio-only**	**Merged (PacBio+Reference)**
Full-Splice Match (FSM)	21,131	55,043
Incomplete Splice Match (ISM)	8777	7585
Novel In Catalog (NIC)	20,769	19,082
Novel Not In Catalog (NCC)	23,161	21,055
Genic Genomic	87	72
Antisense	489	575
Fusion	1,021	947
Intergenic	636	3290

Table S4. Gene Ontology (GO) enrichment terms for biological process for genes with both DIU and Major Isoform Switching between the Active and Hibernating seasons (any gene expression level), FDR < 0.05.

**GO biological process complete**	**Ref.**	**Test**	**Expected**	**Fold Enrichment**	**Raw P-value**	**FDR**
acetyl-CoA metabolic process (GO:0006084)	31	8	1.12	7.11	5.10E-05	1.67E-02
purine nucleoside bisphosphate biosynthetic process (GO:0034033)	55	10	1.99	5.01	8.15E-05	2.37E-02
ribonucleoside bisphosphate biosynthetic process (GO:0034030)	55	10	1.99	5.01	8.15E-05	2.33E-02
nucleoside bisphosphate biosynthetic process (GO:0033866)	55	10	1.99	5.01	8.15E-05	2.29E-02
acyl-CoA metabolic process (GO:0006637)	91	14	3.3	4.24	1.77E-05	7.12E-03
thioester metabolic process (GO:0035383)	91	14	3.3	4.24	1.77E-05	6.94E-03
purine nucleoside bisphosphate metabolic process (GO:0034032)	122	16	4.43	3.62	2.72E-05	9.50E-03
ribonucleoside bisphosphate metabolic process (GO:0033875)	122	16	4.43	3.62	2.72E-05	9.29E-03
nucleoside bisphosphate metabolic process (GO:0033865)	122	16	4.43	3.62	2.72E-05	9.09E-03
carboxylic acid catabolic process (GO:0046395)	216	24	7.83	3.06	4.16E-06	2.04E-03
organic acid catabolic process (GO:0016054)	220	24	7.98	3.01	5.54E-06	2.56E-03
response to starvation (GO:0042594)	199	20	7.22	2.77	9.33E-05	2.53E-02
fatty acid metabolic process (GO:0006631)	323	28	11.72	2.39	5.17E-05	1.66E-02
small molecule catabolic process (GO:0044282)	352	29	12.77	2.27	9.95E-05	2.65E-02
carboxylic acid metabolic process (GO:0019752)	808	66	29.31	2.25	4.54E-09	6.49E-06
oxoacid metabolic process (GO:0043436)	832	66	30.18	2.19	1.34E-08	1.62E-05
organic acid metabolic process (GO:0006082)	854	66	30.98	2.13	3.53E-08	3.96E-05
heart development (GO:0007507)	533	41	19.33	2.12	1.57E-05	6.67E-03
monocarboxylic acid metabolic process (GO:0032787)	491	37	17.81	2.08	6.18E-05	1.94E-02
small molecule metabolic process (GO:0044281)	1547	115	56.11	2.05	1.51E-12	2.96E-09
cellular lipid metabolic process (GO:0044255)	903	62	32.75	1.89	3.87E-06	2.03E-03
lipid metabolic process (GO:0006629)	1156	78	41.93	1.86	3.79E-07	2.84E-04
organophosphate metabolic process (GO:0019637)	833	56	30.21	1.85	2.24E-05	8.17E-03
circulatory system development (GO:0072359)	870	56	31.56	1.77	7.25E-05	2.19E-02
organonitrogen compound catabolic process (GO:1901565)	1029	62	37.32	1.66	1.79E-04	4.26E-02
phosphorus metabolic process (GO:0006793)	1779	107	64.53	1.66	5.17E-07	3.69E-04

cellular catabolic process (GO:0044248)	1615	96	58.58	1.64	3.75E-06	2.03E-03
protein transport (GO:0015031)	1183	70	42.91	1.63	1.28E-04	3.19E-02
phosphate-containing compound metabolic process (GO:0006796)	1758	104	63.76	1.63	1.72E-06	1.08E-03
catabolic process (GO:0009056)	1882	108	68.26	1.58	4.73E-06	2.25E-03
cellular response to stress (GO:0033554)	1519	85	55.1	1.54	1.26E-04	3.29E-02
organic substance catabolic process (GO:1901575)	1587	88	57.56	1.53	1.29E-04	3.17E-02
heterocycle metabolic process (GO:0046483)	2625	144	95.21	1.51	7.26E-07	4.75E-04
organic cyclic compound metabolic process (GO:1901360)	2922	160	105.98	1.51	1.78E-07	1.47E-04
regulation of cellular component organization (GO:0051128)	2359	129	85.56	1.51	4.10E-06	2.08E-03
cellular metabolic process (GO:0044237)	7029	377	254.95	1.48	2.34E-19	3.67E-15
cellular aromatic compound metabolic process (GO:0006725)	2688	144	97.5	1.48	2.78E-06	1.62E-03
nucleobase-containing compound metabolic process (GO:0006139)	2474	132	89.73	1.47	1.09E-05	4.74E-03
cellular nitrogen compound metabolic process (GO:0034641)	3193	169	115.81	1.46	6.29E-07	4.30E-04
biosynthetic process (GO:0009058)	2420	128	87.78	1.46	2.02E-05	7.57E-03
organonitrogen compound metabolic process (GO:1901564)	4787	253	173.63	1.46	1.21E-10	1.89E-07
primary metabolic process (GO:0044238)	6869	356	249.15	1.43	2.31E-15	7.27E-12
organic substance biosynthetic process (GO:1901576)	2354	122	85.38	1.43	9.19E-05	2.53E-02
organic substance metabolic process (GO:0071704)	7404	382	268.55	1.42	1.03E-16	8.11E-13
regulation of protein metabolic process (GO:0051246)	2492	127	90.39	1.41	1.35E-04	3.28E-02
metabolic process (GO:0008152)	7882	400	285.89	1.4	1.26E-16	6.62E-13
nitrogen compound metabolic process (GO:0006807)	6360	321	230.68	1.39	9.05E-12	1.58E-08
organelle organization (GO:0006996)	3360	168	121.87	1.38	1.91E-05	7.30E-03
macromolecule modification (GO:0043412)	2825	141	102.47	1.38	1.26E-04	3.25E-02
cellular protein metabolic process (GO:0044267)	3219	159	116.76	1.36	6.56E-05	2.02E-02
cellular macromolecule metabolic process (GO:0044260)	4442	212	161.12	1.32	1.75E-05	7.23E-03
protein metabolic process (GO:0019538)	3807	181	138.08	1.31	1.28E-04	3.24E-02
macromolecule metabolic process (GO:0043170)	5743	265	208.3	1.27	9.05E-06	4.06E-03
cellular component organization or biogenesis (GO:0071840)	5496	249	199.35	1.25	7.77E-05	2.31E-02
cellular process (GO:0009987)	15074	639	546.75	1.17	1.70E-15	6.69E-12
biological process (GO:0008150)	17698	707	641.92	1.1	6.72E-14	1.76E-10

Table S5. **GO enrichment terms for biological process** for genes with DIU and Major Isoform Switching with expression level changes <20% between the Active and Hibernating seasons, FDR < 0.05.

**GO biological process complete**	**Ref.**	**Test**	**Expected**	**Fold Enrichment**	**Raw P-value**	**FDR**
negative regulation of protein metabolic process (GO:0051248)	1028	34	16.12	2.11	5.64E-05	4.43E-02
nucleic acid metabolic process (GO:0090304)	2007	56	31.48	1.78	2.88E-05	2.83E-02
regulation of cellular component organization (GO:0051128)	2359	65	37	1.76	9.36E-06	1.23E-02
regulation of protein metabolic process (GO:0051246)	2492	67	39.08	1.71	1.53E-05	1.72E-02
nucleobase-containing compound metabolic process (GO:0006139)	2474	66	38.8	1.7	2.14E-05	2.24E-02
cellular component biogenesis (GO:0044085)	2481	65	38.91	1.67	4.72E-05	4.12E-02
organelle organization (GO:0006996)	3360	88	52.7	1.67	1.02E-06	2.30E-03
heterocycle metabolic process (GO:0046483)	2625	68	41.17	1.65	3.54E-05	3.28E-02
cellular nitrogen compound metabolic process (GO:0034641)	3193	78	50.08	1.56	5.53E-05	4.57E-02
cellular component organization or biogenesis (GO:0071840)	5496	128	86.2	1.48	6.69E-07	1.75E-03
cellular metabolic process (GO:0044237)	7029	161	110.24	1.46	1.08E-08	1.70E-04
cellular component organization (GO:0016043)	5298	121	83.09	1.46	4.80E-06	6.86E-03
nitrogen compound metabolic process (GO:0006807)	6360	142	99.75	1.42	1.07E-06	2.11E-03
primary metabolic process (GO:0044238)	6869	153	107.73	1.42	2.72E-07	8.56E-04
macromolecule metabolic process (GO:0043170)	5743	127	90.07	1.41	1.13E-05	1.37E-02
organic substance metabolic process (GO:0071704)	7404	163	116.12	1.4	1.28E-07	6.68E-04
metabolic process (GO:0008152)	7882	170	123.62	1.38	2.44E-07	9.60E-04
cellular process (GO:0009987)	15074	278	236.41	1.18	3.85E-08	3.02E-04
biological process (GO:0008150)	17698	304	277.57	1.1	4.80E-06	8.38E-03

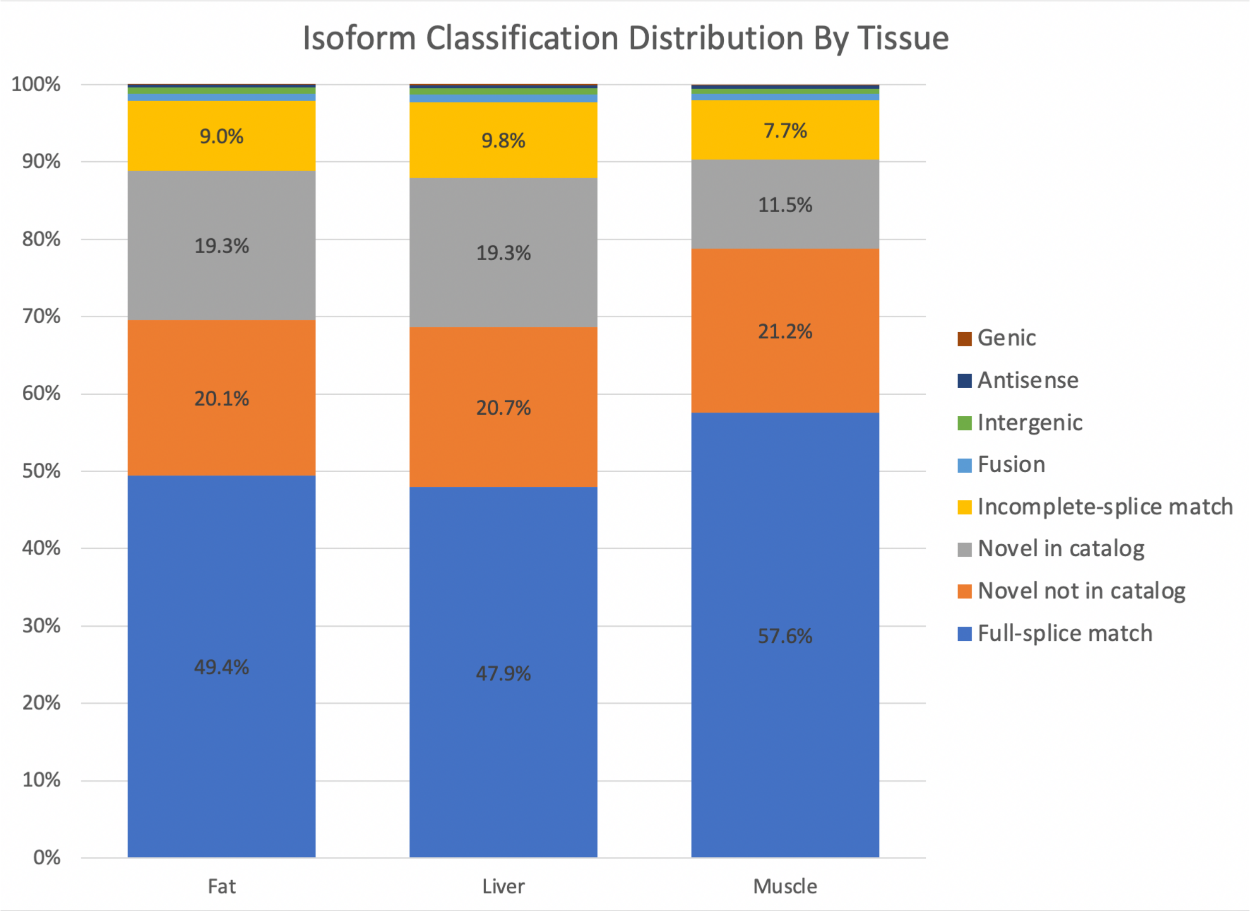

Figure S1. **Transcript Structural Categories** per tissue (a) Adipose (b) Liver (c) Muscle

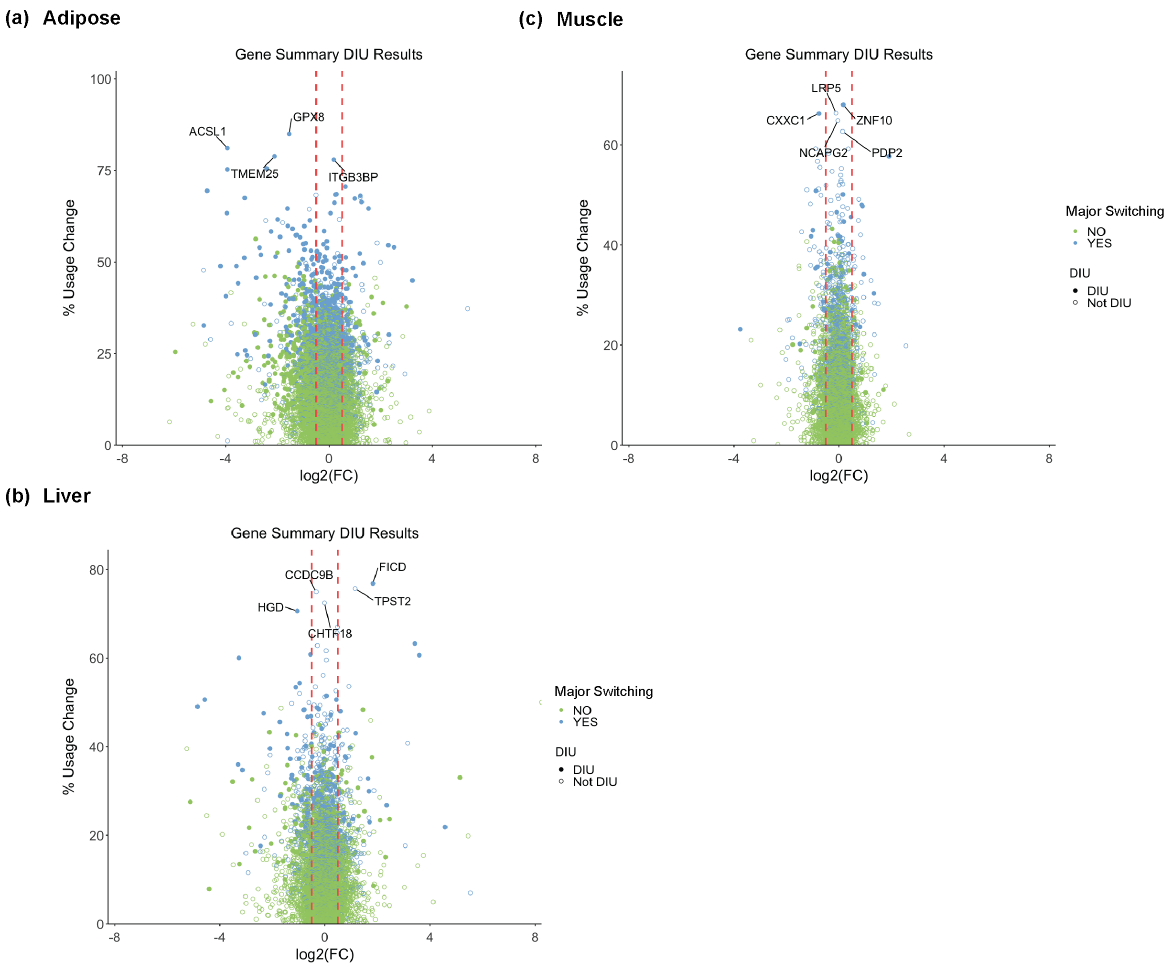

Figure S2. Differential isoform usage and major isoform switching per tissue (a) Adipose (b) Liver (c) Muscle. Percent isoform usage change versus $\log_2$ fold change (log2FC), where colors indicate evidence for major isoform switching (blue indicates major isoform switching) and filled circles indicate evidence for differential isoform usage.

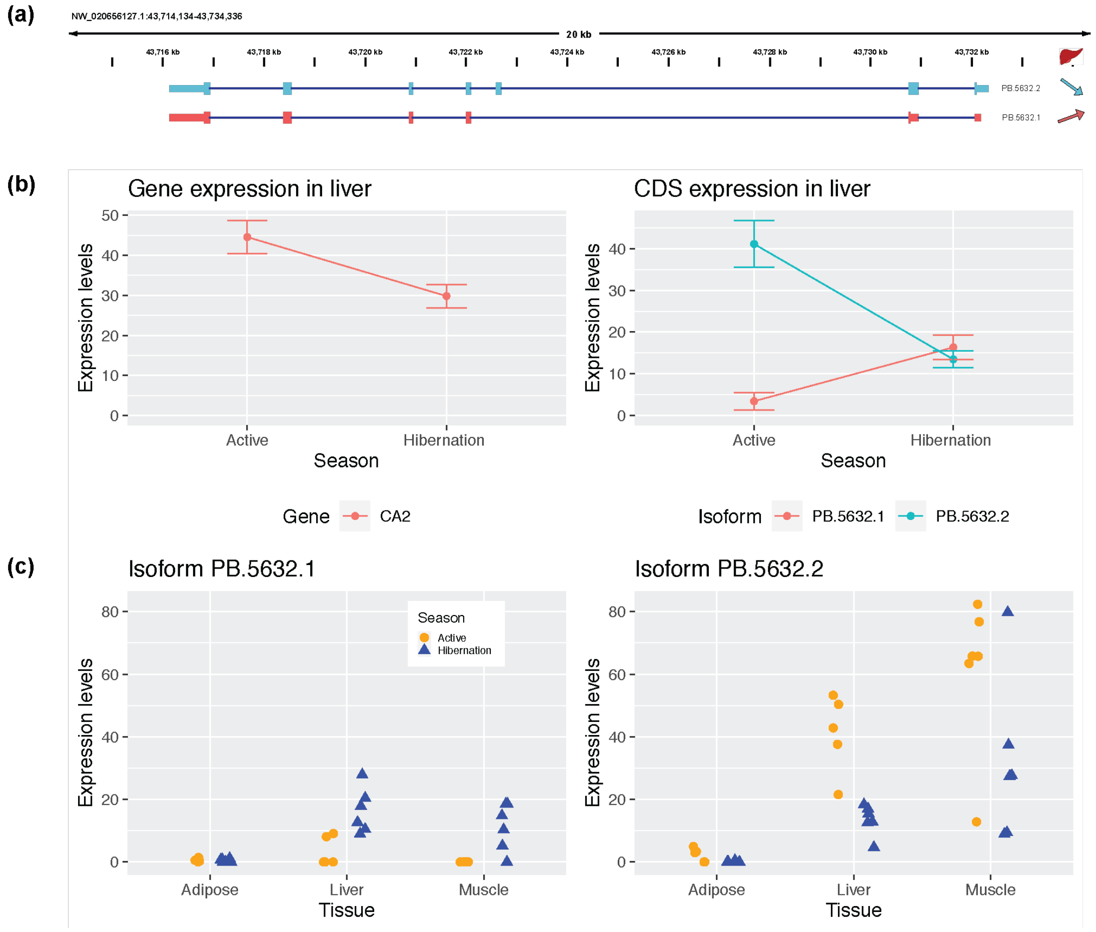

Figure S3. Differential isoform expression of *CA2* mRNA in liver tissue. (a) Isoform structure of two *CA2* isoforms. (b) Expression changes at the gene and coding sequence (CDS) level. (c) Short read expression of the two *CA2* isoforms across the different bears and tissues.

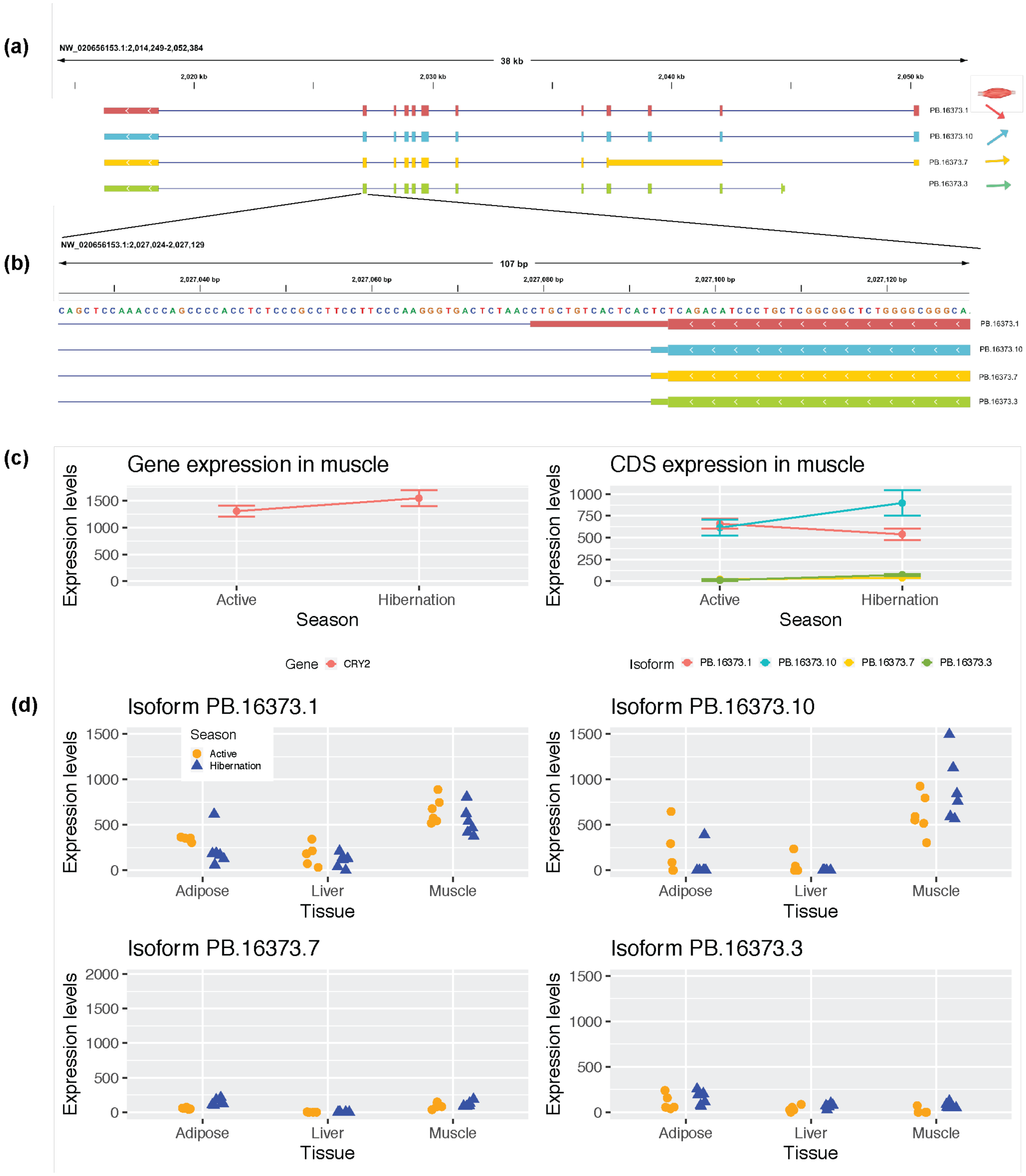

Figure S4. Differential isoform expression of *CRY2* mRNA in muscle tissue. (a) Isoform structure of four *CRY2* isoforms. (b) Zoom in of region that is different between isoforms PB.16373.1 and PB.16373.10 (c) expression changes at the gene and transcript level. (d) Short read expression of the *CRY2* isoforms across the different bears and tissues.

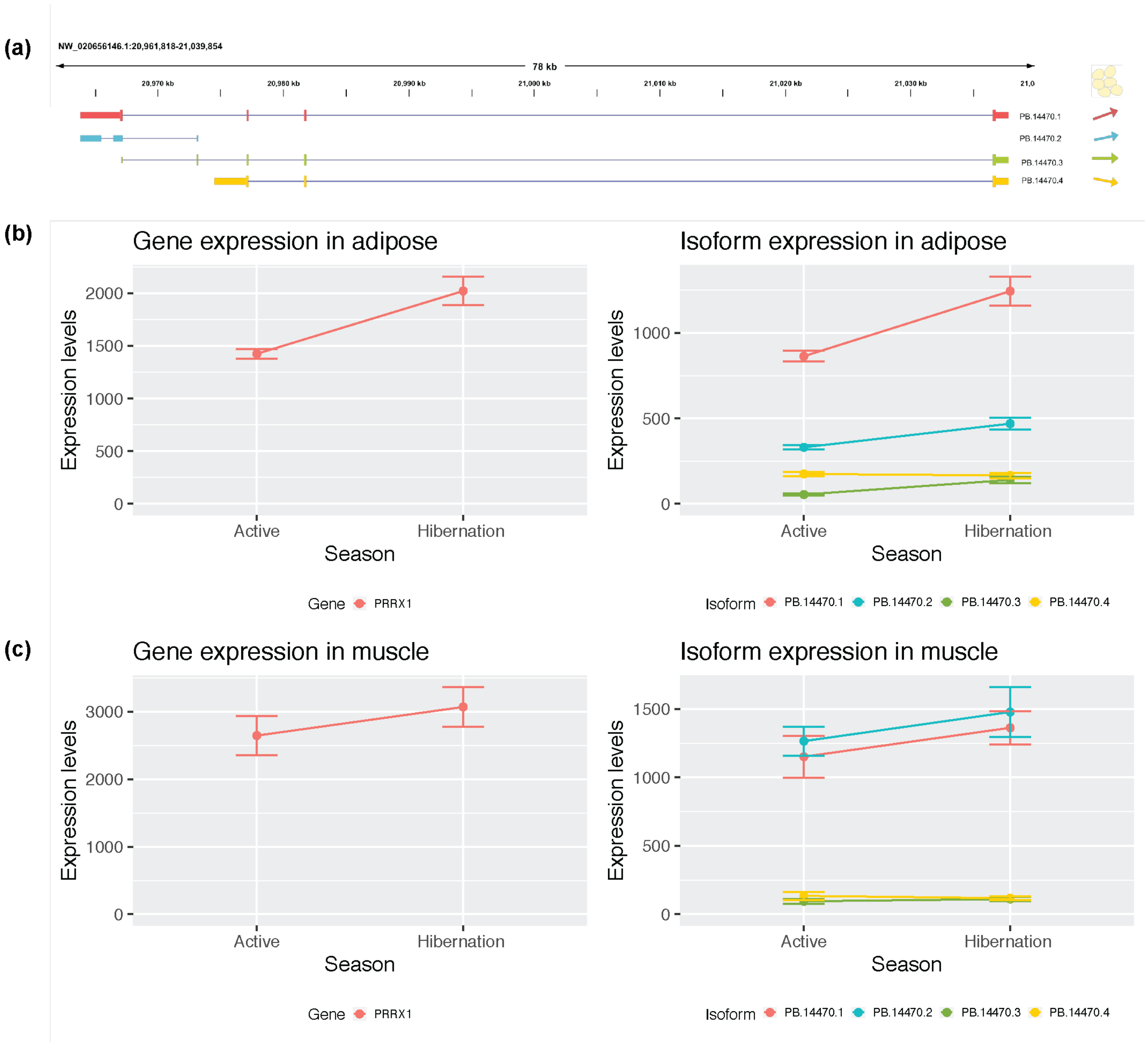

Figure S5. Differential isoform expression of *PRRX1* mRNA in adipose tissue. (a) Isoform structure of four *PRRX1* isoforms. (b) Expression changes at the gene and transcript level in adipose. (c) Expression changes at the gene and transcript level in muscle. Isoform PB.14470.2 (blue) is non-coding.

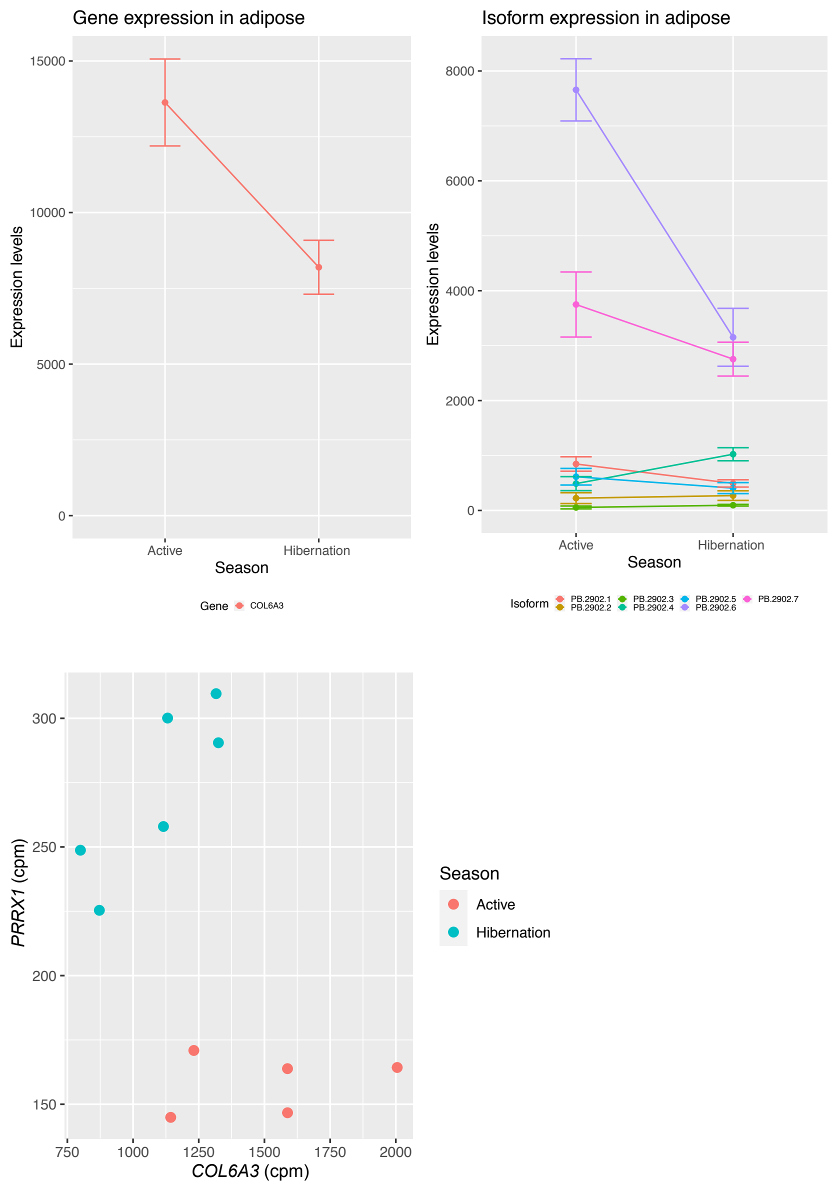

Figure S6. Differential isoform expression of *COL6A3* mRNA in adipose tissue. (a) Expression changes at the gene, transcript, and CDS level in adipose. (b) scatterplot of expression of *PPRX1* mRNA versus *COL6A3* mRNA in adipose, colored by season.
